# Myeloid Sarcoma Type of Acute Promyelocytic Leukemia With a Cryptic Insertion of RARA Into FIP1L1: The Clinical Utility of NGS and Bioinformatic Analyses

**DOI:** 10.3389/fonc.2021.688203

**Published:** 2021-06-24

**Authors:** Yongren Wang, Yaoyao Rui, Ying Shen, Jian Li, Poning Liu, Qin Lu, Yongjun Fang

**Affiliations:** ^1^ Department of Hematology and Oncology, Children’s Hospital of Nanjing Medical University, Nanjing, China; ^2^ Key Laboratory of Hematology, Nanjing Medical University, Nanjing, China; ^3^ Department of Gastroenterology, Children’s Hospital of Nanjing Medical University, Nanjing, China

**Keywords:** acute promyelocytic leukemia, myeloid sarcoma, *FIP1L1–RARA* fusion, next-generation sequencing, *KRAS* gene, bioinformatic analyses

## Abstract

**Background:**

Acute promyelocytic leukemia (APL) is characterized by the presence of coagulopathy at onset and translocation t (15; 17) (q22; 21), meanwhile, other translocation variants of APL have also been reported. The *FIP1L1–RARA* fusion gene has recently been reported as a novel RARA-associated fusion gene.

**Objectives:**

We report a case of *de novo* myeloid sarcoma (MS) type of APL with *FIP1L1–RARA* found by next-generation sequencing (NGS) that was not detected by conventional analyze analysis for RARA translocations.

**Methods:**

We performed typical morphological, magnetic resonance imaging (MRI), conventional tests for PML–RARA dual-fusion translocation probe, high-through sequencing and NGS. Meanwhile, bioinformatics analyses were done by using public repositories, including ONCOMINE, COSMIC, and GeneMANIA analysis.

**Results:**

A 28-month-old girl with a complex karyotype that includes 46,XX,t(4;17)(q12;q22)[9]/46,idem,del(16)(q22)[3]/45,idem,-x,-4,-9,-15,del(16)(q22),+marl,+mar2,+mar3[7]/46,xx[3], c.38G>A (p.Gly13Asp) in the *KRAS* gene, and a cryptic insertion of *RARA* gene into the *FIP1L1* gene was diagnosed with APL complicated by the *de novo* MS.

**Conclusion:**

We report a *FIP1L1–RARA* fusion in a child with APL who presented with an extramedullary tumor in the skull without the classic karyotype using NGS, whom we treated with good results. NGS analysis should be considered for APL variant cases. Further experimental studies to the association between the mutation in *KRAS* gene and *FIP1L1–RARA* fusion on the clinical phenotype and progression of APL are needed to identify more effective therapeutic targets for APL.

## Introduction

Acute promyelocytic leukemia (APL) is a subtype of acute myeloid leukemia (AML) that is cytogenetically characterized by the t (15;17) (q24;q21) translocation and gene fusion between the promyelocytic leukemia (*PML*) and the retinoic acid receptor alpha (*RARA*). Additionally, fewer than 5% of APL patients have other fusion gene between *NPM1*, *NUMA1*, *STAT5B*, *BCOR*, *FIP1L1*, *IRF2BP2*, *FNDC3B*, *PRKAR1A*, *OBCF2A*, *GTF2I* and *RARA*, respectively (1). In 2007, the *FIP1L1/RARA* fusion gene was first reported as a novel *RARA*-associated fusion gene in a patient with juvenile myelomonocytic leukemia (JMML) (2). In addition, it was found in patients with APL in 2008 (3) and 2011 (4). Their fusion gene reported was generated rearrange exon 3 of RARA with exon 15 or 13 of FIP1L1, respectively.

Myeloid sarcoma (MS) in APL is a rare condition and is mainly associated with cases of relapse. Herein, we report a primary MS typing case with *FIP1L1/RARA* that was not detected by conventional tests for RARA-associated translocation.

## Materials and Methods

### Patients and DNA Samples

DNA samples extracted from bone marrow of the patient with APL were sent to the Nanjing Key Laboratory of Pediatrics at the Children’s Hospital of Nanjing Medical University in Nanjing, China, for genetic analysis. These works were approved by the ethics committee of Children’s Hospital of Nanjing Medical University. The patients/participants provided their written informed consent to participate in this study.

### Morphology, Flow Cytometry, and FISH Studies

Cytogenetic analysis at diagnosis was carried out according to a standard procedure on a BM sample processed after short-term culture (24 h). G-banded chromosome was determined according to International System for Human Cytogenetic Nomenclature. FISH analysis was performed using commercially available PML/RARα dual color dual fusion DNA probe (Abbott Molecular, Des Plaines, IL).

### Molecular Analysis

*KRAS* mutation, *FIP1L1/RARA* and the *RARA/FIP1L1* fusion transcripts monitoring were performed by NGS. The size and quality of the pre- and post-captured libraries were evaluated using a 2100 BioAnalyzer (Agilent). The HiSeq X PE 2*150 method was used for sequencing, and each sample was sequenced with 15G PF data.

### Public Database

The summary of the distribution of different types of mutations for *KRAS* mutations in hematopoietic neoplasm was performed using the Catalog of Somatic Mutations in Cancer (COSMIC, release v92, 27th August 2020) database (http://www.sanger.ac.uk/cosmic/) and visualized in the bar chart. All data were extracted on Dec. 2, 2020. ONCOMINE (https://www.ONCOMINE.org) was used to analyze the messenger RNA levels of *KRAS*, *RARA*, and *FIP1L1* in different cancer tissues compared with that in normal control with the significance was generated using Students t-test. Statistically significant values and fold change: P value <1E−4 and fold change >2. The comparison heatmap and boxplot of *KRAS*, *RARA*, and *FIP1L1* expression value across different cancers was generated.

### Statistical Analysis

GraphPad Prism software (version 6, GraphPad Software Inc., La Jolla, CA, USA) and R statistical software (RStudio) were used for statistical analyses. Student’s t-test (two-tailed) was used to compare the means between two groups, mRNA expression data are presented as fold change, and a statistically significant difference was considered at the level of P <0.05.

#### Case Report

**Figure 1:**
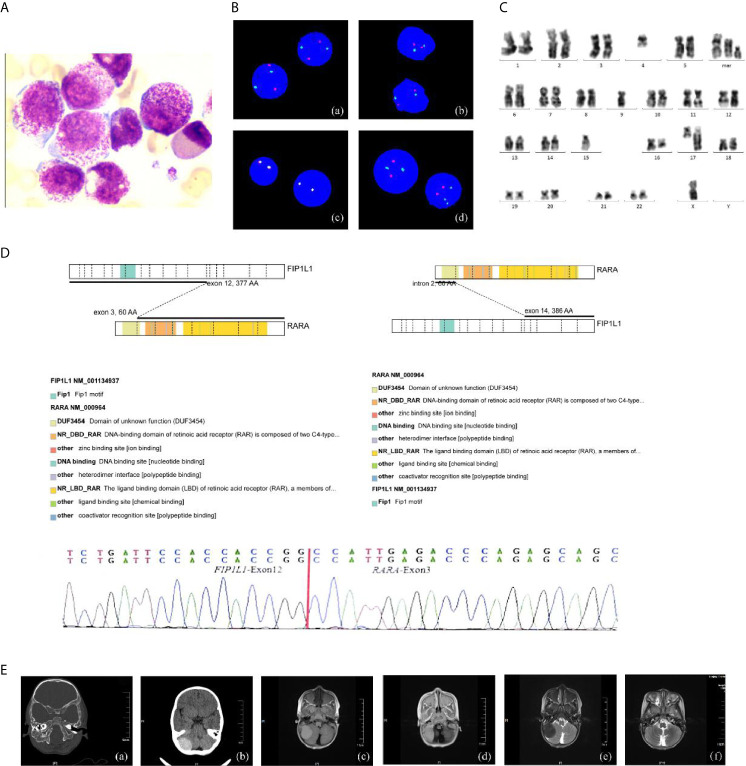
The clinical information of the APL patient. **(A)** Bone marrow morphology at initial diagnosis. ×400; **(B)** FISH using the *PML/RARA* dual-color, dual-fusion translocation probe indicated the absence of the normal *PML/RARA*: (a) nuc ish(D5S23/D5S721,CSF1R)×2[400], (b) nuc ish (D7Z1/D7S486)×2[400], (c) nuc ish (MLL×2) [400], (d) nuc ish (PML×2, RARA×3)[292/400]; **(C)** G-banded karyotype of the patient; **(D)** Sequencing analysis of *FIP1L1–RARA* fusion transcripts atonset. Diagrammatic representation and sequencing information of *FIP1L1–RARA* fusion transcripts. *FIP1L1* transcripts consisting of exon 12 joined to RARA intron 3 (variant form). **(E)** Intracranial tumor: (a, b) CT detects mass with some skull changes in the right posterior fossa, (c–e) MR image: infiltrative lesion involving the right posterior fossa, bilateral mandible, antrum maxillae, skull base, and partial vertebra, (f) complete resolution of the intracranial mass after the treatment.

The 28-month-old girl was presented with pain in her right upper limb. Initial laboratory evaluation presented a high white blood cell (WBC) level of 20 × 10^9^ with 65.2% of abnormal promyelocyte cells, hemoglobin of 9.5g/dl, and a platelet level of 101 × 10^9^/L. Her coagulation function and thromboela-stogram were normal, while lactate dehydrogenase was 740 U/L. The BM aspirate showed 74.5% promyelocytes that had numerous hyper-granularity with Auer bundle; the rate of positive peroxidase staining was 100%, suggestive of APL (**Figure 1A**). The immuno-phenotype was positive for CD4, CD2, MPO, CD33, CD13, CD11b, CD64, CD15, CD71, CD9 and CD65, and negative for CD34, HLA-DR and TDT. Fluorescence *in situ* hybridization (FISH) analysis with the PML–RARA dual-fusion translocation probe identified no dual fusion signal but the presence of increased signals of RARA gene (73%; **Figure 1B**). Besides, cytogenetics revealed a complex karyotype in 19 metaphase cells with the following formula: 46,XX,t(4;17)(q12;q22)[9]/46,idem,del(16)(q22)[3]/45,idem,-x,-4,-9,-15,del(16)(q22),+marl,+mar2,+mar3[7]/46,xx[3] according to ISCN2016 (**Figure 1C**). Mutational analysis of myelogenous leukemia related genes using a high-through sequencing technique showed a KRAS mutation [c.38G >A; p.Gly13Asp with 11.9% variant allele frequency (VAF)] and 28 single nucleotide polymorphisms (SNP) which considered to be no pathogenic significant related to myeloid hematological tumors. To further characterize, we arranged a NGS strategy for hematopoietic malignancies. Sequencing of this sample confirmed *FIP1L1/RARA* and the *RARA/FIP1L1* fusion transcripts. Besides, the fusion gene *FIP1L1–RARA* was generated between exon 12 of *FIP1L1* and exon 3 of *RARA*, while the fusion gene *RARA–FIP1L1* was generated between intron 2 of RARA and exon 14 of *FIP1L1* (**Figure 1D**). This patient was diagnosed with microgranular variant (M3v) of APL according to bone marrow morphology, immunophenotype, cytogenetics, and the transcriptome sequencing.

What’s more, an urgent cerebral computed tomography (CT) scan revealed the presence of high-density shadows in the right posterior fossa with partial skull changes. Then, head and spine MRI showed intracranial mas formation in the right posterior fossa which is considered as MS with extensive infiltrative lesions involving the bilateral mandible, the antrum maxillae, the skull base, and partial vertebra (**Figure 1E**).

After a treatment combining all-trans retinoic acid (ATRA, 20 mg/d, divided twice a day) with DA regimen (daunorubicin [20 mg/d for 5 days] and cytarabine [40 mg/day for 8 days]) as induction chemotherapy, the patient had pain in her eyes on the 14th day of treatment, which was considered to be differentiation syndrome. A second BM smear showed 1.5% blasts, and a second head MR showed that the chloroma had disappeared, indicating that the patient had achieved initial complete remission by day 30. However, FIP1L1/RARA was still positive after the second course. Since November 11, 2020, the patient has received three cycles of ATRA and idarubicin [10 mg/d for 3 days] in the following consolidation treatment. She received continual therapy with ATRA and chemotherapy as previously described and kept leukemia-free after a 5-month follow-up.

#### Bioinformatics Analysis of Genetic Mutations in the *KRAS*

**Figure 2:**
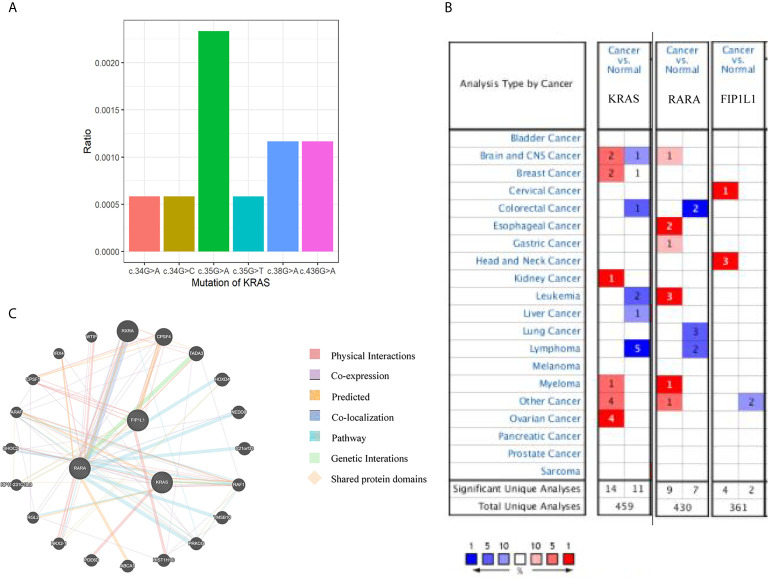
Bioinformatic analyses of *KRAS*, *RARA*, and *FIP1L1* genes. **(A)** An overview of the mutations in *KRAS* in the samples from patients with hematopoietic malignancies, according to the COSMIC database. **(B)** The number of datasets that had mRNA overexpression (red) or down-regulated expression (blue) of the *KRAS*, *RARA*, and *FIP1L1* gene in ONCOMINE. The threshold was designed with the following parameters: p-value of 1E−4 and fold change of 2. **(C)** Network of *KRAS*, *RARA*, and *FIP1L1* and their 20 related genes was analyzed by GeneMANIA.

First, we searched for the mutants of *KRAS* using the COSMIC database (http://cancer.sanger.ac.uk/cosmic): there were mutations in 11 of 1,713 (0.6%) patients in hematopoietic neoplasm, and c.35G >A is the most frequent mutation (**Figure 2A**). We compared the mRNA levels of *KRAS*, *RARA*, and *FIP1L1* among samples of different cancers by using ONCOMINE databases (https://www.ONCOMINE.org). For *RARA*, more datasets showed an increased trend in leukemia patients (**Figure 2B**). For detailed analysis in ONCOMINE datasets, we further searched their expression in APL patients. GeneMANIA was used to analyze associations in terms of co-expression, physical interactions, shared protein domains, predicted, pathway and co-localization among *KRAS*, *RARA*, and *FIP1L1* (**Figure 2C**).

## Discussion

APL is a subtype of AML and is commonly characterized by the expression of the oncogenic PML–RARα fusion protein (more than 95% of APL cases). The incidence of APL in infants is very rare (median age of pediatric APL is 9–12 years) (5). The typical feature of APL is a life-threatening coagulopathy which can lead the patients to death (6). Early deaths (occur in the first 30 days after diagnosis) were associated with a high WBC count (7). The classical diagnosis of APL is represented by the morphology and the flow-cytometry analysis on bone marrow aspirate and confirmed by FISH probes for cytogenetic translocation t (15; 17) or other molecular biology techniques such as reverse transcription PCR for PML–RARA fusion transcript.

Although PML–RARA was failed to be identified by FISH in our case, her abnormal promyelocytes in morphology and immunophenotype were fully consistent with APL, suggesting that her disease may be caused by other X-RARA fusions. In this situation, we use NGS as a tool to discover her fusion gene called *FIP1L1–RARA*. To date, several other partner genes including *ZBTB16*, *BCOR*, *NPM1*, *NABP1*, *Stat5B*, *PRKAR1A*, etc. have been reported (8). Only three cases with cryptic *FIP1L1–RARA* have been reported, one was a 20-month-old boy with JMML (2), one was a 90 year-old woman diagnosed with APL (3), and the last one was a 77-year old female APL patient (4). Both of the two APL patients were old with no DIC. Their fusion gene was respectively generated between exon 15 or 13 of *FIP1L1* and exon 3 of *RARA*, while the reason for two different phenotypes of leukemia caused by *FIP1L1–RARA* is still unknown. Meanwhile, Kondo (3) put forward the reciprocal *RARA–FIP1L1* has none functional role in leukemogenesis. Genetic involvement in APL has clinical value for the choice of the therapy and evaluating prognosis (9). Thus, it is important to combine NGS with karyotype analysis, FISH, and RT-PCR for accurate diagnosis, especially when RARA rearrangements are failed to be identified by conventional methods.

The mutational spectrum of APL differs from other AML subtypes. The molecular feature of somatic mutations in newly diagnosed and relapsed APL is defined by frequent alterations of *FLT3*, *NRAS*, *KRAS*, and *ARID1A/B* genes, and the lack of mutations in non-M3 AML genes (e.g. *DNMT3A*, *NPM1*, *IDH1/2*, and *ASXL1*) (10). *PML–RARA* acts as the main driver mutation in each APL exome. TCGA consortium showed that the expected recurrent somatic mutations in patient with APL were almost twice lower than those in patients with other AML subtypes. Additionally, the number of somatic mutations in leukemia patients was three to seven times lower than in patients with solid tumors (11). Previous studies have reported that the numerous genes involved in APL had similar cell functions, but lacked recurrence and consistency. The impairment of these genes on APL is weaker than the interaction of mutated genes with different functionally related categories (12). Next, it was found that RARA gene and KRAS were co-expressed through PPI network analysis by GeneMANIA. However, the impairment of c.38G >A (p.Gly13Asp) in the *KRAS* gene and the co-expressed *FIP1L1–RARA* fusion APL is not completely clear, further studies have to be performed in the future.

MS is a relatively rare disease lacks specificity in morphology. Our patient with lesions in the head lack of tissue biopsy is one of the important limits. Considering her clinical history, the mass by MRI and CT imaging is very likely extending from the right mandibular region to the skull and the posterior cranial fossa. It needs to be clinically differentiated from hamartomas, abscesses, meningioma, primitive neuroectodermal tumors, Langerhans cell histiocytosis, and so on. Although the bony destruction persisted in the first repeat MRI, there was complete resolution of the Intracranial mass. The initial MRI findings (combined with APL and significant radiological improvement after treatment) confirmed the diagnosis of MS. however, as the tissue biopsy was not performed, residual disease could not be completely ruled out. In addition, the clinical features of MS in our case occur in the absence of coagulation abnormalities that may differ from extramedullary diseases in advanced APL.

MS in APL occurs rarely but frequently at the time of relapse, that involves the skin, lymph nodes, and central nervous system. It has been suggested that MS may be associated with a direct effect of ATRA on adhesion molecules, a consequence of the prolonged survival, a high WBC (>10 × 10^9^/L) at presentation, and the presence of bcr3 *PML/RARA* fusion transcripts (13). Typically, MS may develop *de novo* or concurrently in AML with 2.5–9.11% occurrence (14). Furthermore, MS in AML was enriched with mutations of the RTK-RAS pathway genes (*KRAS*, *NRAS*, *BRAF*, *PTPN11*, and *CBL*) (15), while *KRAS* and *NRAS* mutations occupy 70% (16). The pathogenesis of extramedullary AML tumor is related to the abnormal cellular adhesion molecules and RAS-MAPK/ERK signaling (17). Presently, there are relatively few data for the prevalence of different mutations and mechanisms of extramedullary APL. What’s more, there might be some connection(s) that APL patient with combined *FIP1L1–RARA* and KRAS mutations had a predilection to develop MS.

To the best of our knowledge, there is no consensus on MS treatment (18). Generally, AML-type therapy is effective for *de novo* MS (19). As our patient has APL combined with intracranial MS, we considered the combination of ATRA with conventional chemotherapy (14). In our case, despite the short follow-up, the patient got CR after 1 month course of treatment without recurrence, which emphasized the efficacy of the combination ATRA–chemotherapy therapy on MS. Therapeutic function of ATRA in APL includes activating the gene transcription in myeloid lineage differentiation and degrading the PML–RARα oncoprotein, while arsenictrioxide (ATO) degrades all PML containing molecules and promotes apoptosis. Interestingly, the reported variety of X-*RARA* fusions including *PLZF*, *NuMA*, *NPM*, *STAT5b*, *FIP1L1*, *PRKAR1A*, *ZBTB16*, *OBFC2A*, *TBLR1*, *GTF2I*, *IRF2BP2*, and *FNDC3B* may cause the resistance to ATO treatment due to the lack of ATO binding sites (20).

In conclusion, patients with APL presenting with the fusion gene *FIP1L1/RARA* are rare but have been reported in the literature (4). We found the first case of APL with *FIP1L1/RARA* by using NGS and concurrent MS, while there is no clear treatment guideline so far. The presented patient had achieved complete remission following systemic chemotherapy. Molecular analysis of APL variants is insufficient only through routine analysis, because variant APL has many partners with RARA. We should consider NGS analysis as a conventional method for patients with variant APL. Lastly, further studies are needed to address the cooperation with *FIP1L1–RARA* and *KRAS* in the MS formation of APL.

## Data Availability Statement

The datasets presented in this study can be found in online repositories. The names of the repository/repositories and accession number(s) can be found in the article/supplementary material.

## Ethics Statement

These works were approved by the ethics committee of Children’s Hospital of Nanjing Medical University. Written informed consent was obtained from the individual(s), and minor(s)’ legal guardian/next of kin, for the publication of any potentially identifiable images or data included in this article. Written informed consent to participate in this study was provided by the participants’ legal guardian/next of kin.

## Author Contributions

Patient Management and Data curation: YW, YR, YS, JL, PL, QL and YF. Data analysis YS and JL. Project administration: YW and YF. Writing—original draft: YW and YR. Writing—review and editing: YF. All authors contributed to the article and approved the submitted version.

## Funding

This research was supported by the National Natural Science Foundation of China (81602913, 81670155, 81903383).

## Conflict of Interest

The authors declare that the research was conducted in the absence of any commercial or financial relationships that could be construed as a potential conflict of interest.

## Abbreviations

AML, acute myeloid leukemia; APL, acute promyelocytic leukemia; MS, Myeloid sarcoma; JMML, juvenile myelomonocytic leukemia; WBC, white blood cell; ATO, arsenictrioxide; ATRA, all-trans retinoic acid; CR, complete remission; FISH, fluorescence *in situ* hybridization; NGS, next-generation sequencing, MRI, magnetic resonance imaging, PML, promyelocytic leukemia protein; *RARA*, retinoic acid receptor alpha; COSMIC, Catalog of Somatic Mutations in Cancer; CT, computed tomography; SNP, single nucleotide polymorphisms.
